# Effect of a ward-based outreach team and adherence game on retention and viral load suppression

**DOI:** 10.4102/sajhivmed.v23i1.1446

**Published:** 2022-12-07

**Authors:** Sanele Ngcobo, Steve Olorunju, Tshifhiwa Nkwenika, Theresa Rossouw

**Affiliations:** 1 Department of Family Medicine, Faculty of Health Sciences, University of Pretoria, Pretoria, South Africa; 2 Biostatistics Unit, South African Medical Research Council, Pretoria, South Africa; 3 Department of Immunology, Faculty of Health Sciences, University of Pretoria, Pretoria, South Africa

**Keywords:** HIV, community health workers, HIV, games, retention in care, viral load suppression, AIDS

## Abstract

**Background:**

Only 66% of South African people living with HIV (PLWH) are virologically suppressed. Therefore, it is important to develop strategies to improve outcomes.

**Objectives:**

Assess the effect of interventions on 12-month retention in care and virological suppression in participants newly initiated on antiretroviral therapy.

**Method:**

Fifty-seven clinics were randomised into four arms: Ward-based primary health care outreach teams (WBPHCOTs); Game; WBPHCOT–Game in combination; and Control (standard of care). Sixteen clinics were excluded and four re-allocated because lay counsellors and operational team leaders failed to attend the required training. Seventeen clinics were excluded due to non-enrolment.

**Results:**

A total of 558 participants from Tshwane district were enrolled. After excluding ineligible participants, 467 participants were included in the analysis: WBPHCOTs (*n* = 72); Games (*n* = 126); WBPHCOT–Games (*n* = 85); and Control (*n* = 184). Retention in care at 12 months was evaluable in 340 participants (86.2%) were retained in care and 13.8% were lost to follow-up. The intervention groups had higher retention in care than the Control group, but this only reached statistical significance in the Games group (96.8% vs 77.8%; relative risk [RR] 1.25; 95% confidence interval [CI]: 1.13–1.38; *P* = 0.01). The 12 month virologic suppression rate was 75.3% and was similar across the four arms.

**Conclusion:**

This study demonstrated that an adherence game intervention could help keep PLWH in care.

**What this study adds:**

Evidence that interventions, especially Games, could improve retention in care.

## Introduction

In 2020, an estimated 7 800 000 adults and children were living with HIV in South Africa.^1^ This equates to 20% of the global population of people living with HIV (PLWH). South Africa’s progress towards the UNAIDS 2020 90-90-90 goals were: 90% of PLWH knew their status, 62% of PLWH were on antiretroviral treatment (ART) and 66% of PLWH were virally suppressed in 2020.^1^ An undetectable viral load (VL) predicts normal survival amongst PLWH and virtually eliminates sexual transmission of HIV among HIV-discordant couples. UNAIDS updated their targets to 95-95-95 by 2025, which aim to get more than 95% of PLWH on treatment.^2,3^

Poor adherence to ART is the major cause of therapeutic failure and increases the risk of opportunistic infections and death.^4^ Many factors impede adherence, such as stigma, non-disclosure, unemployment, lack of transport, insufficient access to food, alternative forms of therapy, inadequate follow-up, and lack of patient confidentiality.^5^ In the face of these challenges, there is an urgent need to develop and implement strategies to increase adherence and, subsequently, the proportion of PLWH who attain and maintain an undetectable VL. One such strategy is assigning community health workers (CHWs) to care for PLWH at a household level as ward-based primary health care outreach teams (WBPHCOTs). The role of CHWs in HIV care in sub-Saharan Africa include educating families, caregivers, and communities about HIV/AIDS and its symptoms, as well as preparing PLWH for ART and the possible side effects they may experience.^6^

**FIGURE 1:**
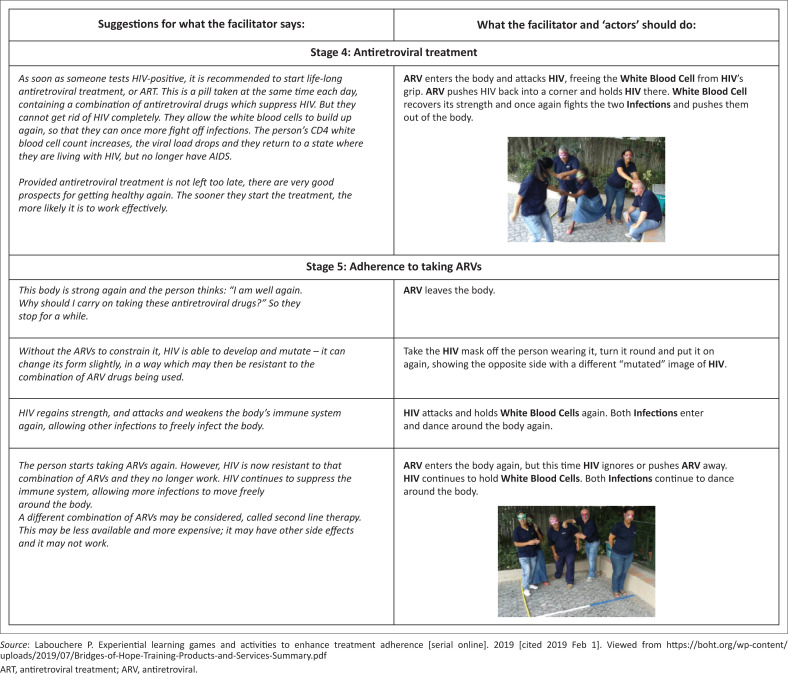
Antiretroviral treatment and adherence game (a description of the antiretroviral treatment adherence game, extracted from the Bridges of Hope’s training guide).

Since low health literacy has been associated with poor health outcomes,^7^ there is growing interest in ways to improve patients’ health literacy in order to improve retention in care and treatment adherence.^8,9^ Traditional ways of patient education have met with limited success^10,11^ and many health disciplines have started experimenting with gamification, where gaming techniques are used to engage audiences and make everyday tasks more fun and engaging. In the HIV field, Bridges of Hope Training developed experiential learning games and activities to enhance treatment adherence (Figure 1^12^). The ART adherence games were designed based on educationally validated theories, such as using experimental metaphor, multisensory learning, social cognitive theory, logical and neurological behavioural frameworks;^13^ and skilled task performance: association, dissociation, and mental models.^14^ The adherence games are specifically designed for use with groups of people who have recently tested HIV-positive, to help them address a range of issues they may face around living positively, such as: stigma, support, disclosure, and treatment adherence. The programme includes 12 sessions, each requiring about 1 hour. Each of the first nine sessions includes two experiential learning activities.

Although both WBPHCOTs and various gaming strategies have shown promise in the health space,^6,15,16^ they have not been tested as a combined strategy in South Africa. To fill this gap, this study assessed the independent and combined effect of a WBPHCOT and games intervention on retention in care and virological suppression of PLWH newly initiated on ART in Tshwane district, South Africa.

## Methods

### Overview

This quasi-experimental interventional study consisted of four arms: WBPHCOTs; Bridges of Hope Training’s adherence game (henceforth referred to as ‘Games’); WBPHCOTs and Games combined; and a Control group that received standard care. Recruitment started in February 2019 and ended in December 2019, and follow-up was done between February 2019 and December 2020. All participants were recruited from primary health care clinics that provide ART. In line with the national programme, newly diagnosed patients were assessed for same-day ART initiation as part of the universal test and treat strategy and visited the clinic monthly (increased to two-monthly once deemed to be stable) to collect treatment.^17^ The sample size was calculated on the assumption that the intervention groups would achieve a 12% improvement in the primary outcome (retention in care), analysed as a binary outcome. Accordingly, a sample size of 460 participants with complete follow-up would give the study an estimated power of 82%.

### Clinic and participant selection

**FIGURE 2:**
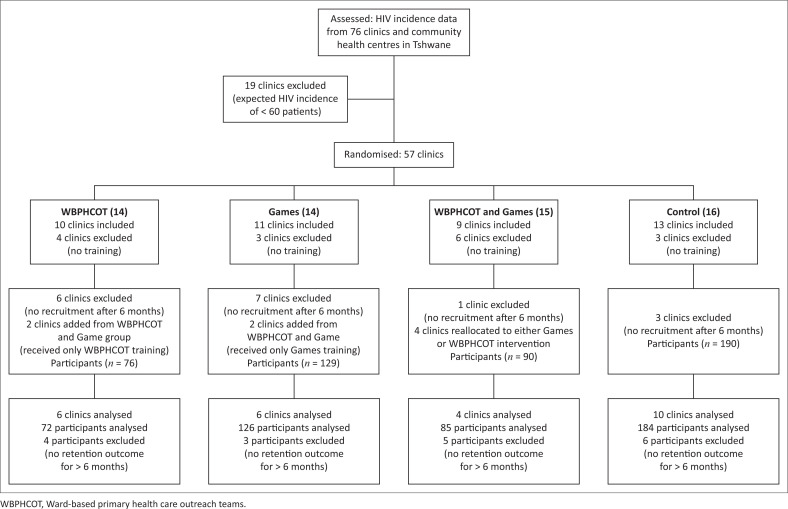
Flow diagram showing number of participants randomised, allocated, followed up and analysed.

Using district health management information systems data, facilities in Tshwane were selected based on the expected number of newly infected persons in their catchment area as calculated from the population size and the national HIV incidence of 1.2%.^18^ Clinics with fewer than 60 potential participants in six months were excluded. Fifty-seven clinics that met the inclusion criteria were randomised in Microsoft Excel into the four study arms. Sixteen clinics were excluded from the study after they were not represented in the required study training. Four clinics were subsequently reassigned to a different intervention since the lay counsellor and operational team leaders failed to attend the required training before patient recruitment began. In addition, 17 clinics that did not recruit participants within the first six months of the study were excluded (Figure 2).

#### Participant inclusion and exclusion criteria

Patients were invited to participate in the study if they were 18 years or older, newly diagnosed with HIV, and initiated on ART within the last month. Patients who were too ill to provide informed consent were excluded. Only participants who were initiated on treatment between January 2019 and December 2019 were included in the study.

### Interventions

Staff that were part of the usual clinic team conducted all interventions in this study. The principal investigator trained staff in preparation for this study. While the integrated access to care and treatment strategy aimed at empowering newly diagnosed PLWH with the skills to manage their condition was introduced in Tshwane at the time, none of the staff members participating in this study was directly involved in it. Staff cadres were similar between the clinics, although case load and responsibilities differed depending on the size of the clinic. All interventions were planned to last 12 months and no tracing strategies were employed for those who missed appointments.

#### Ward-based primary health care outreach teams intervention

Participants from clinics with the WBPHCOT intervention were visited at home by a CHW within a week of treatment initiation. The purpose of the first home visit was to continue with HIV post-test counselling, making use of a patient-centred approach. This session was conducted in a private setting, unless the participant requested members of their family to be present. Counselling was performed by a trained CHW with relevant counselling experience. This session focused on: medical implications of the diagnosis, including prognosis and treatment; disclosure; psychological issues (coping, support, relationships); and social implications (stigma, employment, discrimination). At the end of the first visit, the CHW scheduled a follow-up visit. The second visit focused on treatment adherence support and addressed any arising medical and psychosocial issues. Community health workers also performed a pill count; screened for opportunistic infections, such as tuberculosis, and adverse effects of the medication; and assisted participants with disclosure. After the first two visits, depending on the response of the participant, a CHW did follow-up visits at least once a month, mostly before the date of the participant’s appointment at the clinic. These visits focused on treatment adherence support; checking for, educating about, and managing adverse effects; screening for opportunistic infections, including tuberculosis; providing nutritional advice, mental health care and other services.

#### Games intervention

At least one lay counsellor was trained per facility to offer the Games intervention. The training was conducted over three days by Bridges of Hope Training in collaboration with the Department of Family Medicine of the University of Pretoria. Participants enrolled into this intervention arm had their clinic visits scheduled to coincide with the return dates of at least five other participants in the study. A minimum of six participants played the game at any given time and the game lasted approximately 1 hour. The first game (focusing on treatment adherence) was played approximately one month after initiation of ART and subsequent sessions were scheduled for at least once every month. Each participant was required to play at least the first game to be part of the study. Figure 1^12^ provides a brief description of the Game.

#### Ward-based primary health care outreach teams and Games intervention

This arm consisted of a combination of the two interventions: WBPHCOT and Games. Scheduling occurred in the same way as described previously.

#### Control group

Lay counsellors from clinics allocated to the Control group were trained on the recruitment of participants. Participants in this group received the usual HIV care offered by primary health care clinics. Based on the qualitative evaluation of perceived barriers to offering home-based HIV care by WBPHCOT members in Tshwane,^19^ which reported that CHWs’ role was mainly to trace patients who have been lost to follow-up, it was not expected that the Control group would be exposed to any of the WBPHCOT interventions employed in this study.

### Data collection

All clinics that were part of this study used Tier.net, an electronic version of a paper-based register, with individual patient folders with standardised clinical records captured by data captures. Tier.net consists of individual patient folders with standardised clinical records. It is divided into four parts: the first part contains the patient details, such as folder number, name and surname, date of birth and gender. The second section includes data on ART, including ART start date and tuberculosis treatment started (yes/no). The next section gives details about the outcome: died, transferred, lost to follow-up (LTFU), and retained in care. The last section gives information about the treatment visits, such as pregnancy status, tuberculosis status, VL, and CD4 count; this section is updated every time patients come to collect their medication. Tier.net data for all the clinics were received from the District Information Officer at quarterly intervals. Individual patient folders were used to capture monthly visit data to avoid data being overwritten in the outcome section.

### Outcomes

The primary outcome of the study was retention in care at 12 months. Retention was measured monthly and possible outcomes included: retained in care; LTFU (missed three consecutive clinic visits or confirmed to be untraceable after missing a clinic visit); died; missed a visit; or transferred to another health facility. Outcomes in this study are reported exactly as they were on Tier.net. Participants with retention in care results at 6 months but without a retention outcome at 12 months were recorded under ‘no data’ at 12 months. The retention outcome captured at 12 months was reported as both a binary (retained/LTFU) and categorical (retained/LTFU/died/missed a visit/transferred) variable. We defined retention in care as participants who were retained in facilities where they were initiated. The secondary outcome was virological suppression at 12 months, coded as a binary variable: detectable VL (≥ 50 copies/mL) and undetectable VL (< 50 copies/mL). Clinics taking part in this study monitored VL load 6 and 12 months after treatment initiation, in line with the national HIV guidelines.^20^ Our time window for the 12-month VL was between 9 and 15 months.

### Data analysis

Monthly retention in care data and VL at 12 months were extracted from Tier.net and exported to Microsoft Excel. Data cleaning included identifying and removing duplicates and removing irrelevant observations. Participant characteristics and retention outcome status were analysed using IBM^®^ SPSS^®^ Statistics for Windows, Version 26.0 (IBM Corp., Armonk, New York, United States, 2019).^21^ Results were described according to the mean and standard deviation, frequencies and proportions, as appropriate. Age was analysed as both a continuous, binary (dichotomised according to the mean), and categorical (18–24, 25–34, 35–44, 45+) variable. An intention-to-treat analysis was followed. For the outcomes of interest (retention in care and virological suppression), participants who transferred to other facilities, died, or had no outcome at 12 months were excluded from the analysis. The relative risk (RR) was calculated for retention in care and virological suppression at 12 months, and an odds ratio (OR) was calculated by means of multivariable regression analysis. Participants without VL results at 12 months were excluded from the 12-month VL suppression analysis. All main outcome analyses and planned comparisons defined statistical significance as *P* < 0.05.

### Ethical considerations

The Research Ethics Committee of the Faculty of Health Sciences of the University of Pretoria granted ethics approval for the study (reference number: 580/2018). Permission to collect data was obtained from Tshwane Health district and all facilities involved. Each participant gave informed consent before participating in the study. Patients meeting the eligibility criteria were informed about the study by lay counsellors and gave written informed consent before being enrolled in the study. Participation was completely voluntary, no treatment was delayed, and confidentiality was always respected. The Tshwane Research committee granted the clearance certificate for the study (71/2018) before the study began.

## Results

Of the 558 participants enrolled in the study, 73 were excluded from the analysis as they started treatment before January 2019 and therefore did not meet the inclusion criteria. An additional 18 participants who did not have retention in care data for at least six months at the end of the study were excluded from the data analysis (Figure 2).

### Characteristics of participants

Four hundred and sixty-seven participants from 26 clinics across Tshwane met the inclusion criteria for this study and were included in the analysis: 72 in the WBPHCOT group, 126 in Games, 85 in WBPHCOT and Games, and 184 in the Control group (Figure 2). Of these, 320/467 (68.5%) were female, of whom 64 (20.0%) were pregnant. The WBPHCOT and Games group had the highest proportion of women 62/85 (72.9%) and the Games group had the highest proportion of pregnant women 25/85 (29.4%). The WBPHCOT group had the highest proportion of participants ≥ 35 years old (56.9%), while the Games group had the highest proportion of participants < 35 years old (57.1%) (Table 1). No pre-initiation VL was available since this test is not conducted in the national HIV treatment programme.

**TABLE 1:** Characteristics of participants.

Characteristics	WBPHCOT				WBPHCOT + Games				Games				Control				Total			
	*N*	%	Mean	s.d.	*N*	%	Mean	s.d.	*N*	%	Mean	s.d.	*N*	%	Mean	s.d.	*N*	%	Mean	s.d.
Number of participants	72	15.4	-	-	85	18.2	-	-	126	27.0	-	-	184	39.4	-	-	467	100.0	-	-
Number of clinics per intervention	6	23.1	-	-	4	15.4	-	-	6	23.1	-	-	10	38.5	-	-	26	100.0	-	-
Age Categories			36.60	10.78			36.94	10.99	-	-	33.84	10.93	-	-	34.66	10.95	-	-	35.18	11.00
< 35 years	31	43.1	-	-	40	47.1	-	-	72	57.1	-	-	94	51.1	-	-	237	50.7	-	-
≥ 35 years	41	56.9	-	-	45	52.9	-	-	54	42.9	-	-	90	48.9	-	-	230	49.3	-	-
Gender categories																				
Female	46	63.9	-	-	62	72.9	-	-	85	67.5	-	-	127	69.0	-	-	320	68.5	-	-
Male	26	36.1	-	-	23	27.1	-	-	41	32.5	-	-	57	30.1	-	-	147	31.5	-	-
Pregnant women out of all women	9	19.6	-	-	4	6.5	-	-	25	29.4	-	-	26	20.5	-	-	64	20.0	-	-
Pre-ART CD4 count in cells/mm^3^	-	-	399	270	-	-	362	257	-	-	417	311	-	-	348	282	-	-	381	280

WBPHCOT, Ward-based primary health care outreach teams; ART, antiretroviral treatment.

### Outcome at 12 months

#### Overall outcome

At 12 months, 293 out of the 467 participants (62.7%) remained on treatment in the facility where they were initiated, 18 (3.9%) had no retention data, seven (1.5%) died, 23 (4.9%) had missed a clinic visit, 47 (10.1%) were confirmed LTFU, and 79 (16.9%) had been transferred to another facility (Table 2). There were no statistically significant differences between the intervention and control groups in terms of the number of deaths or missed clinic visits. Only three participants (2.4%) were confirmed to be LTFU at 12 months in the Games group, compared to 28 (15.2%) in the Control group (*P* < 0.001). A higher proportion of participants remained in care in both the Games (72.2%, *P* < 0.001) and the WBPHCOT and Games group (68.2%, *P* = 0.02), compared to the Control group (53.3%). There was, however, no significant difference in retention of participants in the WBPHCOT group (63.9%, *P* = 0.12) when compared to the Control group. A higher proportion of participants were transferred to other facilities in the Control group (21.2%) compared to the WBPHCOT and Games group (10.6%, *P* = 0.03).

**TABLE 2:** Participant outcomes at 12 months.

Characteristics	No data		*P*	Died		*P*	Missed visit*		*P*	LTFU		*P*	Retained in care at the same facility		*P*	Transfer		*P*
	*n*	%		*n*	%		*n*	%		*n*	%		*n*	%		*n*	%	
**Total**	18	3.9	-	7	1.5	-	23	4.9	-	47	10.1	-	293	62.7	-	79	16.9	-
**Intervention**																		
WBPHCOT (*n* = 72)	5	6.9	0.64	1	1.4	0.48	2	2.8	0.58	6	8.3	0.14	46	63.9	0.12	12	16.7	0.42
W+G (*n* = 85)	0	0.0	0.03 **	3	3.5	0.06	5	5.9	0.57	10	11.8	0.46	58	68.2	**0.02**	9	10.6	**0.03**
Games (*n* = 126)	3	2.4	0.19	2	1.6	0.35	8	6.3	0.43	3	2.4	**< 0.001**	91	72.2	**< 0.001**	19	15.1	0.18
Control (*n* = 184)	10	5.4	Ref	1	0.5	Ref	8	4.3	Ref	28	15.2	Ref	98	53.3	Ref	39	21.2	Ref
**Gender**																		
Female (*n* = 320)	12	3.8	Ref	4	1.3	Ref	17	5.3	Ref	32	10.0	Ref	202	63.1	Ref	53	16.6	Ref
Male (*n* = 147)	6	4.1	0.88	3	2.0	0.57	6	4.1	0.58	15	10.2	0.95	91	61.9	0.80	26	17.7	0.77
**Pregnant**																		
No (*n* = 256)	6	2.4	Ref	3	1.2	Ref	12	4.9	Ref	23	9.3	Ref	168	68.0	Ref	35	14.2	Ref
Yes (*n* = 64)	3	4.7	0.33	1	1.6	0.80	6	9.4	0.17	8	12.5	0.45	30	46.9	**< 0.001**	16	25.0	**0.04**
**Age group (years**)																		
18–24 (*n* = 85)	3	3.5	0.45	1	1.2	0.90	6	7.1	0.20	7	8.2	0.38	47	55.3	**< 0.001**	21	24.7	**< 0.001**
25–34 (*n* = 152)	6	3.9	0.40	0	0.0	0.22	11	7.2	0.15	25	16.4	0.01	89	58.6	**< 0.001**	21	13.8	0.16
35–44 (*n* = 130)	7	5.4	0.19	5	3.8	0.18	3	2.3	0.74	10	7.7	0.41	76	58.5	**< 0.001**	29	22.3	**< 0.001**
45+ (*n* = 100)	2	2.0	Ref	1	1.0	Ref	3	3.0	Ref	5	5.0	Ref	81	81.0	Ref	8	8.0	Ref

LTFU, lost to follow-up; WBPHCOT, Ward-based primary health care outreach teams; Ref, reference; W+G, WBPHCOT and Games.

* , Missed visit represents participants who missed their visit at 12 months.

** , Bold figures indicate significant values < 0.05.

There were no significant differences between men and women in any of the outcomes. However, the proportion of pregnant women who remained in care at 12 months was significantly lower than non-pregnant women: 46.9% versus 68.0% (*P* < 0.0001). In addition, a higher proportion of pregnant women were transferred to other facilities when compared to non-pregnant women (25.0% vs 14.2%, *P* = 0.04). The age group 25–35 years had the largest proportion of LTFU (16.4%). In contrast, the age group 45 years and above had the highest proportion of participants who remained in care (81.0%) and were the least likely to be transferred (8.0%).

#### Retention in care

Retention in care outcomes were restricted to binary outcomes, with participants either retained in care or LTFU. At 12 months, 340 participants were either retained in care (86.2%) or LTFU (13.8%) (Table 3). The Games group had the highest retention in care rate of 96.8% and RR 1.25 (95% CI: 1.13–1.38; *P* = 0.01) when compared to the Control group (77.8%). Although proportionally more participants were retained in the other intervention groups, there was not a significant difference in retention rates between the WBPHCOT and the Control group, or the WBPHCOT and Games and the Control group. Younger participants (< 35 years) were significantly less likely to be retained in care when compared to older (≥ 35 years) participants (81.0% vs 91.3%, RR: 0.88, 95% CI: 0.81–0.97, *P* = 0.006).

**TABLE 3:** Factors associated with retention in care, considered as a binary outcome.

Characteristics	Retention in care rate		Relative risk‡	*P*
	*n*	%		
**Intervention**				
WBPHCOT (*n* = 52)†	46	88.5	1.14 (0.99–1.30)	0.10
Games (*n* = 94)†	91	96.8	1.25 (1.13–1.38)	0.01 *
WBPHCOT + Games (*n* = 68)†	58	85.3	1.10 (1.00–1.27)	0.21
Control (*n* = 126)†	98	77.8	Ref	Ref
**Gender**				
Female (*n* = 234)†	202	86.3	1.03 (0.59–1.83)	0.91
Male (*n* = 106)†	91	85.8	Ref	Ref
**Pregnant**				
Yes (*n* = 38)†	30	65.2	0.57 (0.27–1.18)	0.13
No (*n* = 191)†	168	88.0	Ref	Ref
**Age group (years)**				
< 35 (*n* = 168)†	136	81.0	0.88 (0.81–0.97)	**0.006**
≥ 35 (*n* = 172)†	157	91.3	Ref	Ref

WBPHCOT, Ward-based primary health care outreach teams; Ref, reference.

* , Bold figures indicate significant values < 0.05.

† , *n* = combined number of participants retained in care and lost to follow-up;

‡ , Data in brackets represent 95% confidence interval.

**FIGURE 3:**
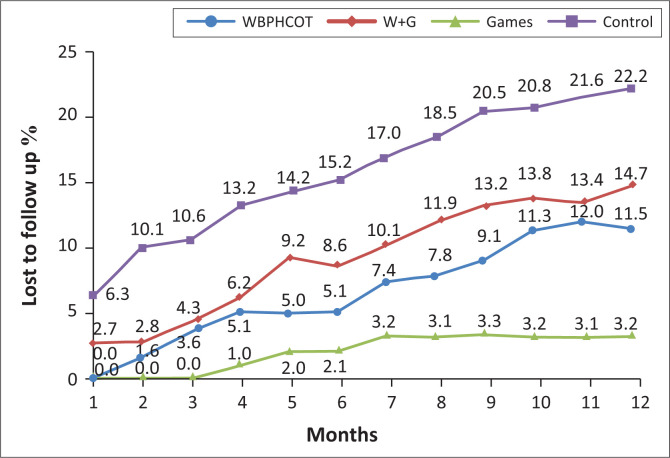
Monthly lost to follow-up rate (according to the binary outcome).

Monthly LTFU data (Figure 3) show that 6.3% in the Control group, 2.7% in the WBPHCOT and Games group, and none in the WBPHCOT group or the Games group were LTFU a month after participants were initiated on treatment. At three months, 10.1% of participants in the Control group were LTFU, compared to 4.3% in the WBPHCOT and Games group and 3.6% in the WBPHCOT group. No participants were LTFU in the Games group at this point. By 12 months, 22.2% in the Control group were LTFU compared with 14.7% in the WBPHCOT and Games group and 11.5% in WBPHCOT group, and 3.2% in the Games group.

#### Virological suppression

At 12 months, 332 out of the 467 participants (71.1%) had their VL results recorded on Tier.net. A similar proportion of participants achieved virological suppression in the three intervention arms when compared to the Control arm (Table 4). Pregnant women had a lower rate of virological suppression than non-pregnant women.

**TABLE 4:** Virological suppression.

Characteristics	VL suppression rate (%)	Relative risk‡	*P*
Overall (*n* = 332)	75.3	-	-
**Intervention**			
WBPHCOT (*n* = 52)	82.7	1.23 (0.67–2.27)	0.49
Games (*n* = 98)	67.3	0.76 (0.57–1.02)	0.08
WBPHCOT + Games (*n* = 68)	76.5	0.95 (0.61–1.47)	0.80
Control (*n* = 114)	78.1	Ref	Ref
**Gender**			
Female (*n* = 237)	77.6	1.14 (0.95–1.36)	0.12
Male (*n* = 95)	69.5	Ref	Ref
**Pregnant†**			
Yes (*n* = 48)	64.6	0.52 (0.32–0.87)	0.01 *
No (*n* = 185)	81.1	Ref	Ref
**Age group (years)**			
< 35 years (*n* = 172)	74.4	0.95 (7.6–1.21)	0.70
≥ 35 years (*n* = 160)	76.3	Ref	Ref

VL, viral load; WBPHCOT, Ward-based primary health care outreach teams; Ref, reference.

* , Bold figures indicate significant values less than 0.05.

† , Three female participants did not have their pregnancy status captured.

‡ , Data in brackets represent 95% confidence interval.

In multivariable logistic regression, only the association between pregnancy and virological failure remained statistically significant, with an OR of 2.37 (95% CI: 1.15–4.9; *P* = 0.019) (Table 5).

**TABLE 5:** Multivariable analysis of factors associated with virologic failure.

Step 1†	Variables in the equation							
	*B*	s.e.	Wald	*df*	Sig.	Exp(*B*)	95% CI for Exp(*B*)	
							Lower	Upper
Pregnant on ART Start	0.862	0.367	5.522	1	0.019	2.367	1.154	4.856
AgeBinary	0.022	0.336	0.004	1	0.949	1.022	0.529	1.972
Intervention	−0.010	0.150	0.005	1	0.946	0.990	0.738	1.328
Constant	−1.437	0.478	9.032	1	0.003	0.238	-	-

ART, antiretroviral treatment, s.e., standard error; *df*, degree of freedom; Sig., significance; CI, confidence interval.

† , Variable(s) entered on step 1: pregnant on ART start, AgeBinary (< 35 years and > 35 years, intervention.

## Discussion

In this study, we found that an adherence game intervention significantly improved retention of participants who had been newly initiated on ART than while previous studies have evaluated digital games for HIV prevention and care,^8,9,22,23^ there is little information on the effectiveness of physical games that aim to improve patients’ adherence to treatment. Physical games could be of special importance in settings where access and knowledge of digital technology are limited.

The overall LTFU rate was 10.1% after 12 months of treatment. When excluding participants who had died, missed visits, were transferred to other facilities, and those without an outcome at 12 months, the LTFU rate was 13.8%. A meta-analysis (2008–2013), published in 2016, reported a 23% LTFU rate at 12 months in 22 studies from South Africa.^24^ This is comparable to the rate in the Control group (22.2%), but almost double that of the WBPHCOT intervention groups and almost seven times more than the Games group in our study.

In a study of 1041 patients who were receiving home-based HIV care from CHWs in Rwanda, 92.3% were retained in care, compared to 88.5% from our study.^25^ Even though the groups exposed to the WBPHCOT intervention outperformed the Control group, the difference in retention in care was not significantly different. This was contrary to our expectation. The researchers therefore conducted a qualitative evaluation of perceived barriers to offering home-based HIV care among WBPHCOT members and PLWH in a different study population.^19^ These included: patients giving incorrect addresses; fear of stigma through association with WBPHCOTs, especially those in uniform; little or no preparation of patients for home-based care; and lack of confidentiality and trust. Future interventions should take these perceived barriers into account in order to optimise the benefit that might be obtained from utilising WBPHCOTs.

We expected the combination of the WBPHCOT and Games intervention to have the best results in terms of retention in care and viral suppression. While this group did indeed have better retention compared to the Control group, it was worse than in the Games group. Furthermore this group had the highest proportion of participants transferred to other facilities. This could have contributed to the lower proportion of participants retained in care in the overall analysis (Table 2). It appears that logistical challenges had made it difficult for CHWs to work with lay counsellors who were offering the Games intervention; this could have negatively affected the outcome in this intervention. Some participants would have preferred one intervention, instead of both. For example, some participants preferred the Games intervention, but refused CHW visits to their households. Some of these challenges were previously noted in community-based HIV prevention trials in KwaZulu-Natal, with some participants refusing to take part in some components of the intervention because of perceived stigma.^26^ This is an important consideration for future intervention studies.

At the time of our study, most participants were on tenofovir, emtricitabine, and efavirenz which has higher pretreatment resistance when compared to the current first-line option (tenofovir, lamivudine, and dolutegravir). Results of patients on tenofovir, lamivudine and dolutegravir may be different from those of this study.^27,28^ The overall rate of VL suppression in our study was 75.3% and was similar among the groups. Pretreatment drug resistance in South Africa is currently at 23.0%, mostly driven by resistance to the non-nucleoside reverse transcriptase inhibitor class.^29^ Since participants in this study would have been initiated on an ART regimen including the non-nucleoside reverse transcriptase inhibitor, efavirenz, it can be expected that a large proportion would fail to achieve virological suppression, despite adequate adherence.^30^

It is interesting that the proportion of participants with virological suppression recorded in our study is lower than the 12-month suppression rates of 90.6% (national) and 92.3% (Tshwane district), reported in adult South Africans in 2018.^31^ It is however important to note that this national and district suppression rate is based on the national indicator data set, which defines suppressed VL as < 400 copies/mL, while in our study we used a definition of < 50 copies/mL.^31^ Pregnant women were significantly less likely to achieve a suppressed VL. Although not directly comparable, a study of 10 052 pregnant women living with HIV reported a 56.2% VL suppression rate, mostly due to late antenatal care booking.^32^ The Games intervention had the highest proportion of pregnant participants; this could have contributed to the high proportion of participants with an unsuppressed VL in this group.

### Limitations

The study is limited by the absence of a randomised design. The quasi-experimental design was necessitated by the inability to ensure training and participant recruitment in all the allocated facilities. The associations identified in this study do, however, meet some requirements of causality, because the intervention preceded the measurement of the outcome. The coronavirus disease 2019 pandemic limited the availability of VL results since the number of clinic visits decreased as per national restrictions. Furthermore, it impacted negatively on the implementation of the interventions, as most CHWs were asked to assist facilities with screening for coronavirus disease 2019 in the community and in health facilities. There were restrictions around gatherings; this affected the arms of the study, especially the Games intervention. However, it is important to note that more than 70% of participants were recruited in the first two months of the study and had therefore not been affected by the pandemic. Barriers to offering home-based HIV care by CHWs negatively affected intervention arms with WBPHCOT members; these barriers were clearly documented in our qualitative study.^19^ Out of 57 randomised clinics, only 26 reached the final stage of data analysis since some clinics failed to attend the required training and some had not recruited any participants by six months. No data were collected on existing adherence support offered in clinics that were part of this study. Only the list of participants who participated in the first game (focusing on treatment adherence) was sent to the principal investigator. Unfortunately, no additional data are available on the number of games played by each participant.

## Conclusion

This quasi-experimental interventional study demonstrated that a physical adherence game improves the retention of PLWH newly initiated on ART and hence has the potential to improve treatment outcomes. Despite the challenges encountered with the WBPHCOT-containing interventions, they still outperformed the Control arm, and the observed improvement might become significant once the potential barriers identified in the study have been addressed. More studies should be performed to assess the optimal way of including WBPHCOTs in home-based HIV care as well as how to combine different interventions in a meaningful way.
